# Differences in mutational processes and intra-tumour heterogeneity between organs: The local selective filter hypothesis

**DOI:** 10.1093/emph/eoz017

**Published:** 2019-06-07

**Authors:** Mathieu Giraudeau, Tuul Sepp, Beata Ujvari, François Renaud, Aurélie Tasiemski, Benjamin Roche, Jean-Pascal Capp, Frédéric Thomas

**Affiliations:** 1 CREEC, UMR IRD 224-CNRS 5290-Université de Montpellier, Montpellier, France; 2 Institute of Ecology and Earth Sciences, University of Tartu, Vanemuise 46, Tartu 51014, Estonia; 3 School of Natural Sciences, University of Tasmania, Private Bag 55, Hobart, Tasmania 7001, Australia; 4 Centre for Integrative Ecology, School of Life and Environmental Sciences, Deakin University, Waurn Ponds, Victoria 3216, Australia; 5 Université de Lille-sciences et technologies, UMR 8198 Evo-Eco-Paleo, Villeneuve d'Ascq/CNRS/INSERM/CHU Lille, Institut Pasteur de Lille, U1019-Unité Mixte de Recherche 8204, Lille, France; 6 IRD, Sorbonne Université, UMMISCO, F-93143, Bondy, France; 7 Departamento de Etología, Fauna Silvestre y Animales de Laboratorio, Facultad de Medicina Veterinaria y Zootecnia, Universidad Nacional Autónoma de México (UNAM), Ciudad de México, México; 8 INSA/Université Fédérale de Toulouse, Laboratoire d'Ingénierie des Systèmes Biologiques et des Procédés, UMR CNRS 5504, UMR INRA 792, Toulouse, France

**Keywords:** cancer, intra-tumoural heterogeneity, selection, mutation

## Abstract

Extensive diversity (genetic, cytogenetic, epigenetic and phenotypic) exists within and between tumours, but reasons behind these variations, as well as their consistent hierarchical pattern between organs, are poorly understood at the moment. We argue that these phenomena are, at least partially, explainable by the evolutionary ecology of organs’ theory, in the same way that environmental adversity shapes mutation rates and level of polymorphism in organisms. Organs in organisms can be considered as specialized ecosystems that are, for ecological and evolutionary reasons, more or less efficient at suppressing tumours. When a malignancy does arise in an organ applying strong selection pressure on tumours, its constituent cells are expected to display a large range of possible surviving strategies, from hyper mutator phenotypes relying on bet-hedging to persist (high mutation rates and high diversity), to few poorly variable variants that become invisible to natural defences. In contrast, when tumour suppression is weaker, selective pressure favouring extreme surviving strategies is relaxed, and tumours are moderately variable as a result. We provide a comprehensive overview of this hypothesis.

Lay summary: Different levels of mutations and intra-tumour heterogeneity have been observed between cancer types and organs. Anti-cancer defences are unequal between our organs. We propose that mostly aggressive neoplasms (i.e. higher mutational and ITH levels), succeed in emerging and developing in organs with strong defences.

Extensive genetic, cytogenetic and epigenetic variation, as well as phenotypic diversity exist between and within tumours (i.e. inter- and intra-tumour heterogeneity, respectively, the latter being called ITH thereafter) [1–4]. Three main types of stochastic phenomena leading to ITH can be distinguished. The most well-known and by far most studied is genetic variability resulting from mutational processes [5]; a more recent but also intensively studied field is epigenetic variability [6] and finally the most largely unexplored is gene expression variability [7]. The implications and clinical importance of these different sources of ITH are considerable since ITH may underlie incomplete treatment responses, acquired and/or innate resistance, and disease relapse in response to chemotherapy and targeted agents [8–12]. Because ITH is an important clinical determinant of patient outcomes, its origins have been the subject of much discussion by investigators. While genomic instability seems to be *the* major proximate process generating ITH [13], no consensus has however emerged between the several (non-mutually exclusive) hypotheses proposed to explain its establishment and maintenance [14, 15] (Box 1). Interestingly, several studies have also highlighted that both mutational processes and ITH between cancer types display a relatively constant hierarchical pattern between organs, with for instance melanoma and lung cancers being on average the most heterogenous cancers [16–19]. Despite extensive research, the processes behind this hierarchy remain unclear as well.

In this paper, we propose that variation in mutational patterns and ITH result from the evolutionary ecology of organs’ theory [20], and are therefore explained by the same rules than those governing mutational patterns and polymorphism in organisms living in more or less adverse habitats [21, 22]. For instance, genomic diversity is generally positively correlated with abiotic and biotic stress levels (e.g. [21, 23, 24]), leading sometimes to the selection of hyper-mutator phenotypes [25, 26]. Beyond a high-threshold level of stress, the diversity may also sometimes decline to a few adapted genotypes potentially displaying strong evolutionary convergence [27, 28]. Organs in organisms can be considered as specialized ecosystems in a living landscape, whose ecologies are more or less favourable to cancer progression. The evolutionary ecology of organs’ theory predicts that the evolution of organ-specific resistance to malignant emergence and/or progression should be governed by their level of exposure to oncogenic factors together with the host’s evolutionary responses in relation with the direct or indirect fitness importance of each organ [20]. Here, assuming that the number of mutations typically found in a cancer is an indicator of the diversity of molecular characteristics of cancer cells, we discuss the extent to which this hypothesis could explain inter organ variability in mutational and ITH hierarchy patterns, as well as examine whether it could explain the predilection for metastatic site(s) by different cancer types.

## THE LOCAL SELECTIVE FILTER HYPOTHESIS

Recently, Vittecoq *et al.* [29] argued that a promising research direction for discovering novel anticancer therapies consist in exploring cancer suppressive mechanisms in animals living in environments that favour cancer emergence and/or progression. Indeed, the same way as the lack of correlation between body size/life expectancy and cancer incidence led to Peto’s paradox [30], a lack of correlation between exposure to oncogenic factors and cancer incidence might suggest that evolution has produced solutions to avoid and/or control malignant problems in those species. From an evolutionary perspective, a similar conceptual framework can be applied at the organs’ level [31, 32]. Concretely, we expect that selection has locally shaped powerful natural defences against malignant emergence/progression, and hence mostly aggressive neoplasms (i.e. higher mutational and ITH levels), or conversely few invisible ones succeed in emerging and developing in organs strongly exposed to mutagenic substances. In contrast, organs that are less exposed to oncogenic factors have been less optimized by selection to be efficient at controlling malignant developments, and as a result, less aggressive neoplasms (i.e. lower mutational and ITH levels) may regularly emerge and progress in these tissues. These predictions seem in accordance with the hierarchical patterns observed for both mutational processes and ITH. Indeed, skin, lung or the digestive tract are, all things being equal, for instance undoubtedly more exposed to mutagenic substances than breast, pancreas or thyroid [33–35]. This phenomenon is of course exacerbated in our modern world [36]. Similarly, differential exposures to injuries and/or to infections, which can promote secondarily carcinogenesis, exist between organs [37].

Following the same idea, it has been long accepted in evolutionary immunology that strong immunological defences are also costly at the organ and tissue levels in terms of oxidative damage, since increased level of reactive oxygen species (ROS) is a by-product of elevated metabolism associated with an immune response, but also a defence mechanism used by immune cells [38, 39]. The level of these oxidative costs (or the strength of protection against these costs) can be organ specific, as has been demonstrated in several studies on wild animals (e.g. [40, 41]). Accordingly, organs that are more efficient at controlling early-stage malignant emergence at the level of immune responses could be more vulnerable to tumour-promoting inflammation and mutations caused by ROS on the genomic level, resulting in higher ITH of neoplasms in these organs. Thus, tumour-promoting inflammation and antitumor immunity coexist at different points along the path of tumour progression, and environmental and micro-environmental conditions should dictate the balance between the two [42].

Carcinogenesis also typically occurs within the spatial constraints of the epithelial layer of the organ. In the breast and pancreas, for example, this involves tumour growth within a narrow duct while in the colon, premalignant lesions (e.g. polyps) grow into the lumen of the bowel and on the skin. The cell–cell interaction network is another factor that could explain the differential sensitivity of organs and tissues to neoplasm development. Indeed, it could be a major contributor among the cancer suppressive mechanisms in animals. When examining the connectivity of 144 cell types in terms of ligands and receptors, recent works found that hematopoietic lineages are outliers because they are far less connected than all other cell types [43]. These lineages are also known to be the less mutated [16] and seem to not necessitate strong genetic instability, suggesting that a major suppressive force might be situated at the level of the cell–cell interaction network. Nevertheless, apart from blood cancers, brain cancers such as glioma can also be characterized by low mutational load. Interestingly, gliomas are also the ones with the highest ITH among solid tumours [19], suggesting that the low mutational load in these cases is compensated by high ITH. As previously discussed (Box 2), if tissue disruption is an initiator event in oncogenesis [44, 45], a strong and dense cell–cell interaction network is expected to more efficiently prevent malignant development. Thus, the more cells are connected in a tissue or an organ, higher ITH is necessary for oncogenesis to occur, at least during the first steps of tumourigenesis. On the contrary, less connected tissues are expected to contribute to cancer types with the lowest ITH levels. Interestingly, works in ecology revealed a correlation between the connectivity between species in an ecosystem and the resistance to invaders [46], suggesting again that homology between species in ecological niches and cell types in organs could be relevant.

The local selective filter hypothesis not only provides an explanation for the different levels of mutations and ITH observed between cancer types and organs, but also supports the fact that cancer could initiate when this selective filter at the tissue and organ levels is broken down. Interestingly, genes linked to multicellularity are systematically repressed in solid cancers while those that are more associated with unicellularity are upregulated [47]. This observation, which is concordant with the atavism hypothesis [48, 49], suggests that cancer cells transit to a more ‘selfish’ unicellular mode of life through an active and directed process driven by selection [48]. Especially, genes linked to the extracellular matrix and adhesion as well as signalling and cell communication are mostly downregulated. Among the seven cancer types studied, those (breast and prostate) that have the most similar expression profile of multicellularity associated genes to normal tissue are also the least mutated [50], suggesting again that the level of genetic instability could be dependent on the need to break down the network of dense cellular interactions.

Finally, the nature and frequency of cancer stem cells are still a controversial debate. Inconsistencies in the numbers of such cells reported in the literature can be a consequence of the different definitions used by researchers. As suggested below (Box 2), oncogenesis could result from tissue disruption that generates differentiation problems because of the lack of tissue control [44, 45]. In our opinion, cancer stem cells have to be considered as cells acquiring highly unstable and variable phenotypes similar the ones of normal stem cells (due to high gene expression noise), but without the normal control normally exerted by the micro-environment.

If cancer development depends especially on the ability to counteract the cell–cell interaction network, it could be assumed that dedifferentiation (from differentiated cells) or failure of differentiation (from adult stem cells) is the best way to generate cells that are no more submitted to micro-environmental control because of the intrinsic plasticity and instability of such cancer stem-like cells [44, 51]. Consequently, the more cells of a tissue are under the control of their environment, the more they would need to acquire stem-cell like properties (and in higher number) to overcome this local selective filter. Tumours in tissues with stronger local selective filter would logically contain more cells with such unstable phenotypes. Thus, this framework could explain the differences in the frequency of cancer stem cells between tumours. As these cells are themselves a source of phenotypic heterogeneity, this would be also associated to higher non-genetic ITH in these tumours.

## METASTATIC PREDILECTION SITE

Due to higher mutation rates, cancerous cell communities originating from neoplasias in organs with high resistance to malignant emergence should be able to produce metastases in a wider variety of organs compared with less diverse tumour cell communities. The understanding that metastasis results when tumour cells interact with a specific organ’s micro-environment stems from the ‘seed and soil’ hypothesis, stating that certain tumour cells (‘seed’) have specific affinity for the milieu of certain organs (‘soil’), and metastases form only when the seed and soil are compatible [52, 53]. Although this hypothesis has been one of the most persistent in the study of cancer, and supported by a wide range of experimental evidence [53], it has not been linked to the mutation and ITH patterns. At the same time, it is logical to assume that the most lethal metastatic ‘seeds’ evolve as a result of selective pressure in the primary tumour [54], and the selective pressure assumed to be the highest in organs that are most protected against malignant developments.

For example, while melanoma (rated highest on mutational processes by [50] and 2nd by [17]) cells introduced in mouse circulation can cause tumour development in a wide variety of tissue types [55], human ovarian cancer (ranked 14th at ITH by [50] and 19th by [17]) cells, despite continuous entry of millions of tumour cells into the circulation, rarely cause metastases even to the lung, the first capillary bed encountered [56]. Similarly, prostate cancer (ranked 15th by [50] and 21st by [17]) ‘seeds’ have a very low probability to find a compatible ‘soil’ in any tissues, but colorectal cancer (1st by [17] and 7th by [50]) ‘seeds’ readily give metastasis in a number of organs, exhibiting a cascadic spread of gastro-intestinal tumours, where metastases in secondary sites spread ‘seeds’ to the organs that follow the blood drain route [57].

**Box 1. Current hypotheses for the establishment and the maintenance of different types of intra-tumour heterogeneity (ITH)**

Several non-mutually exclusive models have been recently published to explain the establishment and maintenance of ITH. For certain cancer/organ combination, it has been argued that a significant proportion of somatic mutations result from **exposures to mutagens**, e.g. ultraviolet light in skin cancers, or tobacco smoking in lung cancers [58]. While this process undoubtedly contributes to generate ITH, it cannot account, alone, for the extreme ITH values frequently observed in certain organs, especially in tiny tumours (e.g. [59]). Waclaw *et al.* [60] proposed a model for tumour evolution suggesting that **cell turnover** together with short-range migration can account for rapid cell mixing within the tumour. Alternatively, according to the **cancer stem cell hypothesis**, ITH results from the differentiation of few cells with stem cell properties (e.g. unrestricted self-renewal abilities) that produce various cell types in the tumour [61]. In parallel, the **linear clonal evolution hypothesis** suggests that ITH is due to the accumulation of various hereditary changes over time that confer selective advantages to some premalignant and malignant cells [62]. Finally, the **plasticity cell hypothesis** postulates that the majority of tumour cells, depending on micro-environmental conditions and/or cell intrinsic stochasticity, display varying degrees of stem cell–like characteristics [14]. In accordance with this idea, Lloyd *et al.* [63] suggested that (at least some) intra-tumour heterogeneity in the molecular properties of cancer cells is governed by predictable regional variations in environmental selection forces. In fact, a common point in these hypotheses is to argue that because ITH plays a crucial role in neoplasia, cancer progression and therapeutic resistance, its persistence, once initiated, is supported by various selective costs and benefits. Although realistic in many cases, this hypothesis has, however, some limitations because environments change unpredictably and evolution cannot anticipate the future. It is, therefore, challenging to explain the occurrence of ITH at the very first steps of the tumourigenesis. Genetic ITH can be so extreme even in tiny tumours, that Ling *et al.* [59] recently argued that evolution under a ‘non-Darwinian mode’ is plausible because genetic diversity observed would be orders of magnitude lower than predicted by simple classic Darwinian selection.

Recently, Thomas *et al.* [15] argued that generative mechanisms of ITH could also provide selective advantages to cells from the first steps of oncogenesis. In this hypothesis, malignant cells achieve greater success by **cooperating** in the process of tumour construction, providing the other with a common good, rather than by just being proliferative in isolation. There would be a concomitant selection of a bet-hedging strategy during oncogenesis, and hence ITH because this is necessary to generate the diversity of cell components needed to build, *de novo*, a novel and an intricate cooperative system like the solid tumour is.

Finally, the molecular heterogeneity within tumours could be fundamentally driven by variations in spatial and temporal distribution of blood flow (see for instance [64, 65]), suggesting that variations in patterns of angiogenesis in different organs could be the primary driver of molecular heterogeneity.

**Box 2. ITH on the level of gene expression**

Apart from genetic ITH, high heterogeneity in gene expression is observed within cancers [7], even at the single-cell level [66]. Cancer cells harbour a continuum of heterogeneous phenotype states demarcated by gradients of marker expression rather than distinct subpopulations [67]. An early increase in non-genetic ITH, especially in gene expression variability from cell-to-cell, has been suggested to account for phenotypic diversification in early steps and ultimately to oncogenesis [44]. Indeed, gene expression variability is modulated during development and differentiation and many studies showed that following a phase of highly stochastic and widespread gene expression, cells progressively transit towards a more homogeneous, coordinated and restricted gene expression pattern [68–70]. Cellular interactions are major determinants in constraining and decreasing gene expression variability and seem to be the main ‘constraints’ leading to these stable differentiated states [71, 72]. For instance, direct cell contacts through gap junctions spatially coordinate prolactin gene expression in pituitary adult tissue [73]. Moreover, enzymatic digestion of extracellular proteins or pharmacological inhibition of gap junctions reduced transcriptional coordination between cells [73], showing that perturbation of cell communication can enhance gene expression variability and phenotypic heterogeneity among differentiated cells.

Thus, tissue disruption could be the initial source of gene expression ITH [44, 45] and genetic instability has been proposed to be caused by this early gene expression ITH [45]. In accordance with this hypothesis, numerous studies have now shown that tissue disruption can be either the inducer or the repressor of the cancerous state [44, 74–76]. Therefore, the presence of epigenetic [77], gene expression [78] or micro-environmental [79] alterations that might precede the emergence of genetically abnormal cells further argues for a major role of non-genetic processes in the first steps of oncogenesis.

The early increase in gene expression variability allows another type of bet-hedging that can synergize with genetic ITH to allow phenotypic diversification, the transcriptional ITH. When RNA-seq data were used to measure the level of transcriptional ITH, 12 major cancer types showed distinct levels of this type of ITH [7]. Interestingly, when these results were compared with previous data on genetic ITH, a positive correlation between genetic heterogeneity and transcriptional ITH was found [7]. Both types of ITH can thus be considered as relevant forces in a bet-hedging strategy where the level of heterogeneity would be dependent on the level of cooperation needed in the process of tumour construction to bypass suppressive forces in tissues.

Finally epigenetic alterations are also increasingly acknowledged as being able to initiate transformation, as genetic alterations do, by providing the gene expression plasticity necessary to provide stochastic oncogenic epigenetic changes [80]. Epigenetic instability can also allow phenotypic diversification in the bet-hedging strategy that we proposed here in the early steps of oncogenesis. Interestingly, while the global levels of genetic and epigenetic variations between tumour types are mostly uncorrelated [81], when epigenetic and genetic ITH were measured by analysis of DNA methylation and copy number alterations in aggressive prostate cancer, the structure of phylogenetic trees constructed from the epigenetic and genetic data were very close, indicating a similarity in evolutionary process [82]. Other works revealed such correlation [6]: for instance, the level of DNA methylation ITH within an individual’s leukaemia was positively correlated with the level of genetic ITH [83]. Landau *et al.* [84] also found this correlation in chronic lymphocytic leukaemia between high numbers of sub-clonal mutations and high DNA methylation ITH. Finally, this correlation between genetic and epigenetic heterogeneity was completed in this last work by an additional correlation identified with data from single-cell RNA sequencing: promoters with high methylation ITH showed high cell-to-cell expression heterogeneity of the corresponding gene [84]. Altogether these works reveal that genetic, epigenetic and gene expression ITH are mostly correlated and suggest that different levels of all these types of ITH, and thus different levels of bet-hedging, are needed depending on the tissue and organ considered. However, despite a diversity of hypotheses that explain why ITH is omnipresent, the reasons governing these different levels of ITH are still unclear.

## CONCLUDING REMARKS

We only see cancers that succeed in their development and this fraction corresponds to malignancies that bypass our natural defences. As soon as defences are unequal between the different parts of the body, the fraction of successful cancers is also expected to vary accordingly. By suggesting that the efficiency of natural defences against cancer development is organ specific and that it explains the hierarchy in mutation load and ITH between organs, our hypothesis highlights the role of the cellular environment in shaping the tumoural genomic and epigenomic content. In standard models of cancer, the cellular environment is destroyed as a consequence of cancer progression, thus has also a passive role and no driver role in tumour evolution. On the contrary, our hypothesis considers that the level of diversity needed for cancer development is precisely dependent on and the consequence of the ability of an organ to suppress this development. It contributes to the continuously growing body of works that place major influences in oncogenesis at higher levels of organization than the genomic level while not denying the major contribution of genetic and epigenetic instability in cancer progression.

An evolutionary theory needs to consider more than just humans, and needs to consider ‘historical’ cancer patterns that would be more in line with the selective pressure experienced by our ancestors (which largely determined our genetic makeup and tumour suppressive strategies). While historical cancer data are undoubtedly difficult to find, we encourage scientists to explore our hypothesis using different datasets among various animal species. We also encourage researchers to perform experimental studies specifically designed to test whether cells with high mutation rate are, as proposed here, more likely to metastasize than others.

## FUNDING

This work is supported by the ANR (Blanc project TRANSCAN) and by the CNRS (INEE).

**Conflict of interest:** None declared.
